# The Family Talk Intervention Improves Family Communication and Psychosocial Health Among Families in Pediatric Palliative Care: A Pre-Post Evaluation Study

**DOI:** 10.3390/children13040471

**Published:** 2026-03-28

**Authors:** Kerstin Ivéus, Maja Holm, Kristofer Årestedt, Ulrika Kreicbergs, Lena Anmyr, Camilla Udo, Malin Lövgren

**Affiliations:** 1 Department of Health Care Sciences, Marie Cederschiöld University, 116 28 Stockholm, Sweden; 2 Institution for Nursing Science, Sophiahemmet University, 114 86 Stockholm, Sweden; maja.holm@shh.se; 3 Faculty of Health and Life Sciences, Linnaeus University, 352 52 Växjö, Sweden; kristofer.arestedt@lnu.se; 4 Institute of Child Health, University College London, Great Ormond Street, London WC1N 1EH, UK; u.kreicbergs@ucl.ac.uk; 5 Social Work in Health, Department of Health Professionals, Karolinska University Hospital, 171 64 Solna, Sweden; lena.anmyr@regionstockholm.se; 6 School of Health and Welfare, Dalarna University, 791 88 Falun, Sweden; cud@du.se

**Keywords:** family talk intervention, psychosocial, family-based intervention, pediatric palliative care

## Abstract

**Highlights:**

**What are the main findings?**

- FTI improved family communication, family satisfaction, parenting skills, and anxiety levels among family members;
- FTI showed reductions in children’s mental health problems.

**What are the implications of the main findings?**

- FTI contributes to pediatric palliative care by providing evidence-based practice that can improve psychosocial outcomes for all family members;
- FTI enhances family communication and psychosocial health. This supports the integration of structured, family-based interventions into pediatric palliative care.

**Abstract:**

Background: The psychosocial needs of families involving a child with a life-limiting or life-threatening condition are well recognized. However, evidence-based interventions that address the needs of the entire family remain scarce, even though family health can be maintained and supported if interventions encompass each individual family member, as well as the family as a unit. The aim was to evaluate the family talk intervention (FTI), regarding family communication, and psychosocial health, for families involving a child with a life-limiting or life-threatening condition. Methods: This pre-post study without a control group involved families of children with a life-limiting or life-threatening condition receiving FTI at a pediatric hospital and a hospice in Sweden. The study is registered at clinicaltrials.gov (ID NCT05020158, date of registration: 23 August 2021). FTI is a family-based intervention with the goal of facilitating family communication about illness-related topics, e.g., prognosis, support parenting, and making all children’s needs visible. In total, 105 participants from 29 families were included. Surveys measuring self-assessed family communication and satisfaction, anxiety, resilience, parenting skills, and children’s mental problems were answered at three time points: baseline (before intervention), at the end of the intervention, and six months later. Changes over time were analyzed using linear mixed-effects models. Results: Significant improvements were reported in family communication, family satisfaction, parenting skills, and levels of anxiety over time. Children’s mental health problems were reduced over time regarding emotional symptoms, conduct problems, peer relationship difficulties, and hyperactivity. No changes were found regarding resilience. Conclusion: The results suggest that FTI contributes to improved family communication and psychosocial health for families involving a child with a life-limiting or life-threatening condition. This highlights the value of a systemic approach that actively involves all family members.

## 1. Introduction

Although the psychosocial needs of families in pediatric palliative care are well recognized, evidence-based interventions that address the needs of the entire family remains scarce [1,2,3,4]. Families of children with life-limiting or life-threatening conditions, as defined by Benini, Papadatou [5], face complex psychosocial needs, where curative and palliative approaches often coexist and can last for several years. The care can span across home, hospital, and hospice settings. The availability of pediatric palliative care varies significantly across regions, with some areas offering local or regional teams, while others lack formal services. These challenges highlight the importance of effective communication within the family and in collaboration with healthcare [6].

Family members describe the importance of open communication that follows the child’s illness trajectory, provides space to discuss illness-related topics, and sustains hope within the family [6,7,8]. Mutual silence, where parents and children refrain from sharing concerns in an attempt to protect one another, constitutes a well-documented barrier to effective communication. It is associated with increased insecurity and confusion among children and may undermine relational trust within the family, underscoring the need for open and developmentally attuned communication [6].

The family remains central to children’s emotional and psychosocial development; the well-being of all family members is deeply intertwined and a key predictor of children’s overall psychosocial health [9,10,11]. Recognizing the family’s central role in children’s psychosocial health also underscores the importance of collaboration with healthcare professionals, as parents rely on them for clinical, emotional, and psychosocial support [12,13]. This interconnectedness stresses the relevance of interventions that support family processes rather than focusing solely on individual family members.

Family-based interventions can promote resilience by strengthening children’s agency in the family and healthcare, by facilitating communication and improving mutual understanding of family members’ needs. Respectful communication within the family may act as a protective factor in the development of anxiety and depression, highlighting its relevance as a key target for interventions aimed at improving children’s psychosocial health [1,14,15,16]. Against this backdrop, interventions specifically designed to strengthen family communication and shared understanding become particularly relevant.

The family talk intervention (FTI) is a psychosocial, family-based intervention originally developed for psychiatric settings when a parent has affective disorder, and is grounded in the rationale that preventive interventions must target influenceable family processes, such as communication, shared understanding of the illness, and children’s meaning-making, to reduce risk transmission and promote resilience [17,18]. FTI has undergone rigorous evaluations in the psychiatry context, where it has been shown to improve family functioning and promote children’s mental health [15,19,20,21]. The core components and mechanisms of change in FTI align closely with the psychosocial challenges described by families in pediatric palliative care, including emotional withholding, uncertainty about how to talk with the child, and difficulties maintaining a shared understanding of the illness.

In line with the Medical Research Council (MRC) framework for developing and evaluating complex interventions [22], FTI has also undergone essential feasibility and pilot testing in palliative care contexts, including families with a severely ill parent and minor children as well as in pediatric oncology [23,24]. These preparatory studies, according to MRC s’ framework, have demonstrated its feasibility and the potential benefits such as enhanced family bonding, communication, and long-term sustainability [24]. Although previous pilot studies have shown promising results, the MRC framework emphasizes that further evaluation is required in clinical practice. The present study was, therefore, designed as a pre-post study and conducted in routine clinical practice, with the aim of evaluating FTI in terms of its impact on family communication and the psychosocial health of children living with life-limiting or life-threatening conditions and their families.

## 2. Methods

### 2.1. Design

This study uses a pre-post design in a clinical setting without a control group. It is derived from a larger effectiveness implementation hybrid design project [25] and is registered at clinicaltrials.gov (ID NCT05020158). The present study focuses on the pre-post evaluation [26] among families involving children with life-limiting/threatening illnesses. The implementation process is described elsewhere [27,28].

### 2.2. The Family Talk Intervention

**Figure 1:**
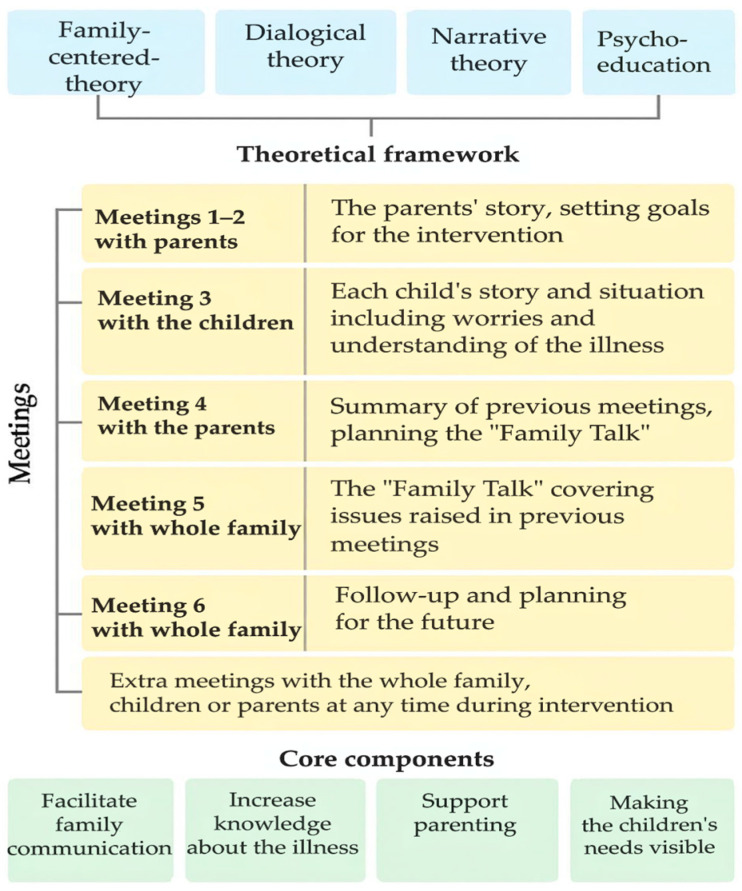
Overview and content of the meetings in the family talk intervention, theoretical framework, and the intervention’s core components.

FTI is a manual-based psychosocial intervention that consists of six meetings with parents and children, alone or together, at different time points (Figure 1). Extra meetings can be added if required. FTI is structured around four core components aiming to foster open communication about the illness and its implications, ensure that children’s needs are acknowledged, and strengthen parenting. The intervention is grounded in an eclectic theoretical framework that combines psycho-educative, narrative, and dialogical methods. The psycho-educative component aims to enhance knowledge about the illness and related topics, while the narrative aspect encourages family members to share personal experiences and collaboratively construct a joint story. The dialogical approach seeks to address problematic situations by amplifying children’s voices and needs, fostering open communication, and highlighting diverse perspectives within the family. These components are theorized to activate mechanisms of change that enable families to navigate illness-related challenges together, strengthen relational bonds, and enhance cohesion and mutual understanding. In turn, these processes are expected to promote resilience and improve psychosocial health outcomes across the family system [15,17,18,29].

### 2.3. Sample and Procedure

FTI was conducted at two pediatric settings: a university hospital and a hospice. The settings were staffed and offered 24/7 services. To participate in FTI, the families were required to have a child with a life-limiting or life-threatening condition, according to the definitions provided by Benini, Papadatou [5]. A minimum of at least two family members were required to participate. The family members defined who belonged to the family.

FTI represented a new practice at the clinics. The healthcare social workers received a ten-session training period prior to delivering the intervention, including practicing FTI under supervision [27,28]. The FTI manual was slightly adapted before it was used, as the original version included ill parents. The manual’s mode of address was revised and the relationships between children were incorporated.

For the study protocol of the present project, a sample size calculation for a dependent-samples t-test (two-tailed) was conducted for the main outcome, family communication. The analysis was based on findings from a previous study using the same instrument [24] and on the minimum required difference reported for the family communication scale (FCS) [30]. With 80% power and a 5% significance level, the calculation indicated that 20 participants were required. After accounting for potential attrition and missing data, it was estimated that 30 individuals needed to be included.

**Figure 2:**
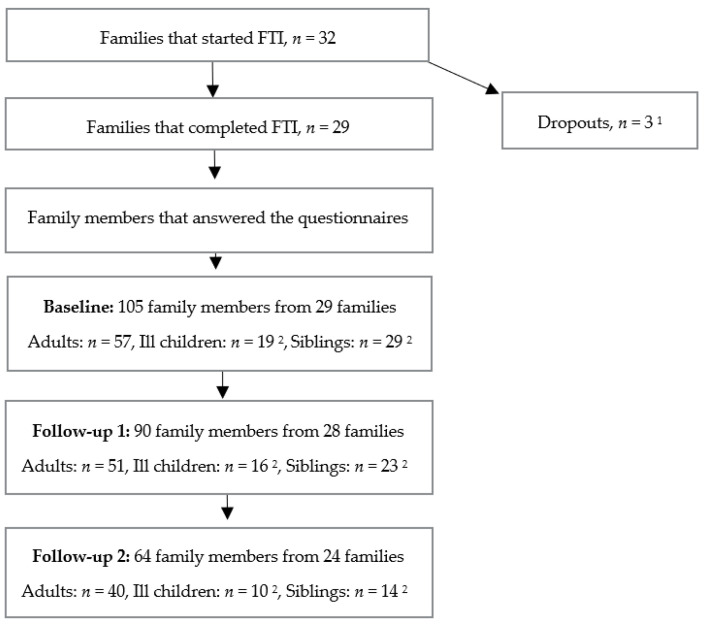
Flow chart over participation and drop-outs. ^1^ One healthcare social worker was on sick leave, thus the family contacted a private therapist instead; one family dropped out for an unknown reason; and one family, a bonus father and siblings, did not complete due to the extended intervention time. ^2^ The total number of ill children or siblings who took part in the intervention was higher than those who completed the questionnaires, due to their condition, age, or for choosing not to participate in the questionnaires.

FTI was conducted from April 2022 to December 2024. Thirty-two families started FTI; three families dropped out during the intervention (Figure 2).

In total, 29 families participated, comprising 57 adults and 48 children. In accordance with national regulations, informed consent was obtained from all participating parents. Children aged 15 years and older provided their own written consent, while younger children gave verbal assent in addition to parental consent. This procedure ensured that participation was voluntary and that all family members were adequately informed about the study.

The study was approved by the Swedish Ethical Review Authority (No. 2020-06341 and 2022-01949-02).

Ten FTI-educated health care social workers identified and recruited eligible families, according to the definition provided by [5]. They provided written and oral information to the families and after obtaining consent, they conducted FTI as part of their everyday clinical work. Because recruitment was embedded in everyday clinical practice, the number of families who were screened or who declined participation was documented in some instances but not maintained systematically by all healthcare social workers.

The healthcare social workers used the FTI manual and a logbook that they completed for each family. The logbook contains all the different components included in the various sessions. When each component was carried out, the healthcare social worker checked it off as completed. The completed logbooks were collected and reviewed by the researcher. This procedure was used as an indicator of fidelity to the intervention, ensuring that core components in the six sessions were implemented as intended.

The contents of each session were personalized to the family’s specific needs. These adaptations were made by the healthcare social workers in dialogue with the family; for example, the intervention was occasionally delivered via video format when appropriate or with a parent present during the child session, if this was the child’s preference. In addition, the healthcare social workers sometimes used creative materials, such as clay or crayons, to help the child feel more secure and comfortable during the session.

### 2.4. Data Collection

Web-based questionnaires were administered at three time points: baseline (pre-intervention), immediately post-intervention (follow-up 1), and six months after follow-up 1 (follow-up 2). The surveys were distributed to the email addresses of the parents and/or children. Within two weeks, adult family members received up to three reminders via text message or telephone to either complete the questionnaire themselves or assist their child, if they had not responded.

#### 2.4.1. Outcome Measure

The outcome measures were chosen based on the goals of the FTI and the mechanisms of change that the intervention aims to provide (Table 1).

#### 2.4.2. Primary Outcome: Family Communication

Family communication was assessed using the Family Communication Scale (FCS; Prepare/Enrich LLC, Roseville, MN, USA) and was answered by participants ≥11 years. FCS is derived from the Family Adaptability and Cohesion Scale IV (FACES IV) [31]. It assesses the perceived ability for effective and open communication within the family and the exchange of information, ideas, thoughts, and feelings among family members [32].

#### 2.4.3. Secondary Outcome: Psychosocial Health

Family satisfaction was assessed using the Family Satisfaction Scale (FSS; Prepare/Enrich LLC, Roseville, MN, USA) for participants ≥11 years. It is also derived from FACES IV [31]. FSS assesses the perceived family satisfaction regarding overall functioning and flexibility;

Parenting was assessed using the Parental Skills Checklist (PSC developed by Lewis [33]; no commercial publisher; used with permission). It is designed to assess the self-assessed interactional behavior the parents use to handle and discuss their children’s illness-related concerns. It comprises two subscales, connecting and coping skills (PSC-CC) and elicitation skills (PSC-ES) [33];

Generalized anxiety disorder was assessed by using the Generalized Anxiety Disorder Scale (GAD-7; Pfizer Inc., New York, NY, USA) [34] for children ≥16 years and adults;

Resilience was assessed using the Resilience Scale (RS 14 and RS10; RS-14; The Resilience Center™, Billings, MT, USA), which is designed to evaluate five central dimensions of resilience: self-reliance, purpose, equanimity, perseverance, and authenticity [35,36]. RS 14 was used on participants ≥13 years, RS-10 was answered by children ≤12 [37];

Children’s mental health was assessed using the Strengths and Difficulties Questionnaire (SDQ; developed by Robert Goodman; Youthinmind, London, UK). It comprises five subscales: prosocial behavior, conduct problems, hyperactivity/inattention, peer relationship problems, and emotional symptoms. For children aged ≤15, the self-report version was used, while a proxy version was applied for children aged ≤10 [38].

### 2.5. Data Analysis

#### Statistical Analyses

Missing-item responses for the outcome measures were imputed using each individual’s median score across the respective scale. This approach was selected because median-based imputation is robust to skewed item distributions, preserves each participant’s response pattern, and avoids assumptions about the underlying score distribution. Item-level missingness was low across the measures in which missing data occurred.

On the FCS, four missing values across three individuals corresponded to 4.4% missing data (4/90 possible item responses). On the FSS, five missing values across three individuals corresponded to 5.6% missing data (5/90). On the PSC, eight missing values across four individuals corresponded to 8.3% missing data (8/96), and on the GAD-7, seven missing values across four individuals corresponded to 8.3% missing data (7/84). No missing values were observed for the RS14, RS10, or SDQ.

Across all measures in which missing data occurred, a total of 24 item responses were imputed using the within-person median approach. As the linear mixed-effects models used in the main analyses appropriately accommodate missing scale-level data across time points, the item-level imputations served only to reconstruct complete scale scores for participants with minimal within-scale missingness. Given the low levels of missing data and the robustness of median-based imputation, this method was considered appropriate and unlikely to introduce meaningful bias.

Descriptive statistics were used to present the baseline characteristics and the outcome measures, including means and standard deviations for continuous variables and frequencies and proportions for categorical variables.

Changes in all outcome variables over time were analyzed using linear mixed-effects models (LMM). The models included random intercepts at two-levels, individuals and families, to account for the dependency introduced by the repeated measurements within individuals as well as the clustering of multiple individuals within the same family. All available observations were included, according to the intention-to-treat principle. Time was specified as a categorical fixed effect to estimate changes across the measurement occasions. Random intercepts accounted for the non-independence of repeated measurements within individuals and the clustering of individuals within families. Attempts to include random slopes for time did not converge, likely due to the limited sample size for some outcome variables. LMM handles missing data under the missing-at-random assumption, allowing for the inclusion of participants with incomplete follow-up data.

Statistical significance was set at *p* < 0.05. Data analyses were performed using IBM SPPS Statistics for Windows, version 25 (IBM Corp., Armonk, NY, USA) and R for Windows, version 4.5.1 (R Foundation for Statistical Computing, Vienna, Austria), including the following packages: lme4 1.1–37 and lmerTest 3.1–3.

## 3. Results

### 3.1. Participants Characteristics

A total of 105 participants were included at baseline (Table 2): 29% mothers, 25% fathers, and 2% other significant adults, 19% ill children, and 28% siblings. The mean age was 44.5 years (SD = 7.2) among adults and 10.9 years (SD = 3.9) among children. In total, 68 children were included among the participating families. Of these, 48 met the inclusion criteria for answering the surveys. Most of the children were classified within the first group of conditions (for example, cancer).

### 3.2. Intervention Effects

Significant changes over time were observed for all outcomes except for resilience, measured by RS14 and RS10, as shown in Table 3.

#### 3.2.1. Family Communication

Family members reported significantly higher levels of perceived family communication (FCS) at the first follow-up (estimate = 1.16, 95% CI [0.01, 2.31]) and the second follow-up (estimate = 1.90, 95% CI [0.61, 3.19]) compared to the baseline.

#### 3.2.2. Family Satisfaction

Family members reported significantly higher levels of perceived satisfaction (FSS) with their families at both the first (estimate = 1.41, 95% CI [0.3, 2.53]) and second follow-up (estimate = 2.57, 95% CI [1.32, 3.82]) compared to baseline.

#### 3.2.3. Parenting

Parents reported significantly higher perceived parental skills (PSC) at the second follow-up (estimate = 3.44, 95% CI [0.28, 6.56]) compared to baseline, but there was no significant change between the first follow-up and baseline. A similar finding was observed for connecting and coping skills (PSC-CC), with higher scores at the second follow-up (estimate = 2.74, 95% CI [0.22, 5.24]). In contrast, no significant change over time was observed for elicitation skills (PSC-ES).

#### 3.2.4. Generalized Anxiety Disorder

Family members reported significantly lower levels of perceived generalized anxiety (GAD-7) at both the first (estimate = −2.0, 95% CI [−3.12, −0.88]) and second follow-up (estimate = −2.38, 95% CI [−3.61, −1.13]) compared to baseline.

#### 3.2.5. Resilience

No significant changes were observed at the first or second follow-up compared to baseline for either RS10 or RS14.

#### 3.2.6. Children’s Mental Health

Children (proxy and self-reports) reported significantly lower levels of perceived mental health difficulties (SDQ-total difficulties) at the first follow-up (estimate = −1.53, 95% CI [−2.90, −0.15]) compared to baseline. This was not sustained in the second follow-up. In contrast, no significant change over time was observed for the pro-social scale (SDQ-Pro social).

## 4. Discussion

This is the first study to evaluate FTI among children with a life-limiting or life-threatening condition and their families in clinical practice. We observed statistically significant improvements in the primary outcome of family communication, as well as in most secondary outcomes, including family satisfaction, parental skills, anxiety, and children’s mental health problems. Despite the lack of a control group, these findings suggest that the intervention may have positive improvements in the context studied.

The findings align with previous studies of FTI in psychiatric settings, which demonstrated improvements in family-functioning and reductions in children’s mental health problems [20], while also decreasing parental anxiety and strengthening family functioning, key factors linked to positive child outcomes over time [21]. Our findings extend this evidence to pediatric palliative care, suggesting that these systemic benefits can be transferable across care settings.

Improved family communication is particularly relevant, as communication is a well-established protective factor during periods of severe stress [39]. This is clinically important, given that open and honest communication has been shown to reduce children’s anxiety and help parents to more accurately understand and respond to their child’s emotional needs, thereby supporting more stable family interactions during the illness trajectory [6]. Findings from this study align closely with the core components of FTI, which emphasize strengthening communication to start the process to enhance family functioning. Moreover, the observed improvements regarding family satisfaction, which capture family functioning in terms of emotional closeness among members, the family’s ability to manage stress, and its flexibility in adapting to changes, indicates strong fidelity to the intervention model and its intended mechanisms of change.

In this study, self-assessed parenting skills had increased at the second follow-up after participation in FTI. This aligns with Giannakopoulos, Solantaus [21], who found that both FTI and a parent-only intervention improved parenting, although overall family functioning improved more, and more quickly, with FTI. Our study extends these findings to pediatric palliative care, where families face exceptional emotional and practical challenges. Including children in FTI appears critical for fostering open communication and mutual understanding, thereby enhancing family functioning and perceived parenting competence. By providing space for children’s voices in line with the United Nations Conventions on the Rights of the Child (UNCRC) [40], FTI not only supports the acknowledgement of the child perspective, but also strengthens family dynamics and promotes parental engagement, benefiting both parents and children. Moreover, by actively involving fathers, FTI may promote equity in parenting and support a balanced family dynamic, which is important for family cohesion and children’s mental health [41].

The improvement observed regarding anxiety is clinically relevant. This finding aligns with previous research, showing that nearly one-third of mothers to severely ill children suffered from clinically significant anxiety [42]. Given that our study demonstrated a reduction in anxiety that persisted at the six-month follow-up, FTI appears to provide sustained symptom relief beyond the immediate post-intervention period. Early interventions targeting anxiety have been associated with a reduced risk of later emotional and behavioral problems [43]; generalized anxiety symptoms in adolescents are linked to social difficulties at school [44]. Furthermore, parental generalized anxiety disorder has been associated with an increased long-term risk of anxiety problems in children [45]. In this context, the anxiety reduction observed in our study suggests that FTI may help to counteract intergenerational transmission of anxiety, which underscores its potential clinical value beyond the immediate intervention period.

The positive changes in children’s mental health problems were found at the first follow-up but not at the second. This result contrasts the results seen in Giannakopoulos, Solantaus [21], where improvements in family functioning and the associated positive effects on children’s mental health were maintained over time. However, it is important to consider the heterogeneity of the sample of children. The study included both children with life-limiting and life-threatening conditions in the pediatric palliative care context and their healthy siblings. In such a context, it is not unexpected that health outcomes are not stable over a six-month period. For children with life-threatening conditions, there is no inherent expectation that psychosocial or physical health will improve over time; siblings may also experience varying levels of distress. The lack of an overall improvement may, therefore, reflect the clinical reality of pediatric palliative care rather than the lack of improvement as a result of the intervention. Another reflection is that the initial benefits for children may be temporary unless reinforced over time. One possible explanation is that improved parenting and family communication provide an immediate buffer for children’s distress. Repeated or extended sessions of FTI that include the children could, therefore, be necessary to consolidate these relations.

In our study, the resilience measure did not show any improvement; however, the instrument used to measure resilience in children demonstrated low internal consistency, suggesting that this assessment had low reliability. This raises the possibility that the scale may not have adequately captured changes in resilience within the context of a family-focused intervention or among children living with life-limiting or life-threatening conditions and their siblings. It also raises questions about whether individual resilience is the most relevant outcome for family-based interventions. The theoretical foundation of FTI emphasizes systemic processes rather than isolated individual traits [17]. Resilience was included as an outcome because FTI’s theoretical framework assumes that strengthening family-level protective processes supports children’s adaptive functioning. From this perspective, resilience may emerge as a family-level phenomenon as well as an individual attribute in the child. Similarly, previous research has shown that resilience appears difficult to influence at the individual level within short interventions [1]; Furlong, McGuinness [20] found no overall improvements in coping self-efficacy (resilience) after FTI. Taken together, both the limited reliability of the child-resilience instrument and the theoretical orientation of FTI suggest that individual child resilience may not be the most suitable outcome for evaluating this type of intervention. Therefore, future research should prioritize family-level resilience [46] rather than individual measures, which better reflects the relational mechanisms.

A major strength of this study is the inclusion of all family members, including ill children aged six years and older and their siblings, which enables the capture of diverse perspectives and reflects the systemic and relational nature of pediatric palliative care. By engaging the entire family, the intervention addresses complex relational and emotional dynamics, enhancing its relevance and potential impact [47].

It should be noted that the sample size calculation reported in the study protocol was originally conducted for a dependent samples t-test. In the present study, the protocol was not fully followed, as linear mixed-effects models were deemed more appropriate for handling dropouts and to account for the dependency introduced by the repeated measurements within individuals, as well as the clustering of multiple individuals within the same family. The use of linear mixed-effects models strengthened the internal validity of the analyses by accounting for individual variability over time and by incorporating all available data, including partially observed trajectories, while also modeling the nested structure within families [48]. However, it cannot be excluded that the present study was underpowered. The limited sample size also restricted the complexity of the models that could be estimated; for example, random slopes did not converge and, therefore, reduced the generalizability.

Several limitations should also be considered when interpreting our findings. First, while improvements were observed over time, the absence of a control group limits causal inference. We cannot rule out the possibility that the observed improvements would have occurred even without the intervention. External factors, such as natural recovery, regression to the mean, or concurrent influences, may have contributed to the observed changes [49]. Second, the sample size decreased over time due to attrition, which limited the possibility of conducting subgroup analyses that could have provided deeper insight into variables such as gender or type of family member.

Future research should, therefore, include a control group and a sample size that enables subgroup analyses to explore variation across different family constellations and relational patterns. Third, although validated instruments were used, some scales (family communication and satisfaction) lack formal validation in Sweden or for children, which may affect measurement outcomes. Finally, according to the definition by Benini, Papadatou [5], the global population of children requiring palliative care is predominantly composed of those with life-limiting conditions, particularly progressive neurological or metabolic disorders, whereas life-threatening conditions constitute a smaller proportion. In our study, 72% of the participants had life-threatening conditions (Group 1), resulting in a diagnostic distribution that differs from the broader population. This should be considered when interpreting the generalizability of the findings. Recruitment and delivery of the intervention were managed by the healthcare social workers, reflecting their usual role in clinical practice. This dual role may have influenced participation, as families with higher motivation or resources could have been overrepresented, introducing potential response bias. The recruitment process may also have introduced selection bias, as families who agreed to participate might differ systematically from those who declined, for example, with respect to motivation, the perceived need for support, or available psychosocial resources.

Although fidelity was defined as completing all six sessions according to the logbook, some variation in attendance, format, and session content occurred due to clinical and family-related circumstances. These adaptations reflect the responsive tailoring that is often necessary in routine clinical practice and can be viewed as supporting the feasibility of implementing FTI in real-world settings, while still maintaining its core components.

## 5. Conclusions

Although the design does not allow for causal conclusions, the study provides preliminary evidence on the real-world evaluation of FTI in the context of pediatric palliative care. The findings showed improved family communication and satisfaction, reduced levels of anxiety, improved parental skills and reduced mental health problems among children. This highlights the value of a systemic approach that actively involves all family members. Taken together, by engaging children living with a life-limiting or life-threatening condition and their parents and siblings in structured sessions, FTI adopts a whole-family approach that goes beyond current guidelines, which often focus on individual relationships. This approach addresses a critical gap in family-centered pediatric palliative care highlighted in previous research.

In line with MRC guidance on evaluating complex interventions, this real-world clinical study provides an important step; future research should build on these findings by using robust study designs, i.e., randomized trials, and larger samples.

## Figures and Tables

**Table 1:** Overview of outcome measures, target age group, scoring method, and internal consistency (Cronbach’s α) at baseline.

Outcome	Respondents	Number of Items and Scales	Type of Scales	Scoring	Cronbach’s Alpha at Baseline
Primary outcome					
Family Communication Scale (FCS)	≥11 years	10 items, one total scale	5-point Likert-type-scale, where 1 corresponds to “very dissatisfied” and 5 to “very satisfied”	A total score ranging from 10 to 50 points *: 44–50 = very high (highly positive communication) 38–43 = high (generally positive, few concerns) 33–37 = moderate (satisfactory, some concerns) 29–32 = low (multiple concerns) 10–28 = very low (substantial challenges) *	0.87
Secondary outcome					
Family Satisfaction Scale (FSS)	≥11 years	10 items, one total scale	5-point Likert-type scale, where 1 corresponds to “very dissatisfied” and 5 to “very satisfied”	A total score ranging from 10 to 50 points * 45–50 = very high satisfaction 40–44 = high satisfaction 36–39 = partial satisfaction 30–35 = some dissatisfaction 10–29 = substantial dissatisfaction *	0.89
Parental Skills Checklist (PSC)	Parents	8 items, one total scale and two subscales (Connecting and Coping skills, Elicitation Skills)	6-point numeric rating scale, anchored at 0 “never” and 5 “always”	A total score ranging from 0–40, higher scores reflect higher perceived skills	0.93
Subscale Connecting and Coping skills	Parents	6 items	-	A total score ranging from 0–30, higher scores reflect higher perceived skills	0.94
Subscale Elicitation Skills	Parents	2 items	-	A total score ranging from 0–10, higher scores reflect higher perceived skills	0.89
Generalized Anxiety Disorder (GAD–7)	≥16 years	7 items, one total scale	4-point Likert-type scale, where 0 corresponds to “Not at all” and 3 to “Nearly every day”	A total score 0–21 scores 0–4 reflects minimal levels of generalized anxiety 5–9 reflects mild levels of generalized anxiety 10–14 reflects moderate levels of generalized anxiety 15–21 reflects severe levels of generalized anxiety	0.88
Resilience Scale 14 (RS 14)	≥13 years	14 items, one total scale	7-point Likert-type scale, where 1 corresponds to “strongly disagree” and 7 “strongly agree”	A total score ranging from 14–96 * 82–98 high resilience 65–81 moderate resilience 14–64 low resilience	0.84
Resilience Scale for Children (RS-10)	≤12 years	10 items, one total scale	4-point Likert-type scale, where 1 corresponds to “not at all like me” and 4 to “ a lot like me”	A total score ranging from 10–40 * ≥35 indicates higher resilience	0.54
The Strengths and Difficulties Questionnaire (SDQ)	Self-reports from children 11–15 years Proxy for children ≤10 years	25 items (5 subscales: prosocial scale, conduct difficulties scale, hyperactivity scale, peer difficulties scale, and emotional difficulties scale)	-	-	-
Total Difficulties Score		20 items (4 subscales: conduct difficulties scale, hyperactivity scale, peer difficulties scale, and emotional difficulties scale)	3-point Likert-type scale where 0 corresponds to “not true” and 2 to “certainly true”	A total score 0–40 * Self-reports version: 0–15 = Normal (emotional/behavioral difficulties) 16–19 = Borderline 20–40 = Abnormal Proxy version: 0–13 = Normal (emotional/behavioral difficulties) 14–16 = Borderline 17–40 = Abnormal	0.73
Prosocial Score		5 items (1 subscale: prosocial scale)	-	A total score 0–10 * Proxy version Scores: 6–10 = Normal Pro-social behavior 5 = Borderline 0–4 = Abnormal, (indicating low prosocial behavior) Self-reports score: 6–10 = Normal pro-social behavior 5 = Borderline 0–4 = Abnormal (indicating low prosocial behavior)	0.77

* Scoring according to present manual.

**Table 2:** Sociodemographic characteristics of participating families (*n* = 29) and family members (*n* = 105) at baseline.

Type of Family Member, *n* (%)	
Adults	
Mothers	30 (29)
Fathers	25 (24)
Other significant adults	2 (2)
Children ^1,2^	
Ill child	19 (18)
Siblings	29 (28)
Age, mean (SD) [Min-Max]	
Adults	44.5 (7.2) [31–61]
Children	10.9 (3.7) [6–18]
Educational level (adults), *n* (%)	
University	34 (60)
Upper secondary school	17 (30)
Primary school	0
Other	6 (11)
Living with a partner (adults), *n* (%)	
Yes	52 (91)
No	5 (9)
Child’s condition, *n* = 29 ^3^ (parental report), *n* (%)	
Group 1, Serious illnesses that might be cured, but treatment can fail e.g., cancer, heart anomalies	21 (72)
Group 2, Conditions where treatment can prolong life e.g., Sickel cell anemia	3 (10)
Group 3, Progressive diseases with no cure, where care focuses only on symptom relief and may last many years e.g., X-ALD, Tay-Sacchs	3 (10)
Group 4, Permanent but non-progressive conditions causing severe disability, e.g., asfyxia	2 (7)

^1^ Four of the children’s SDQs were answered by a proxy. ^2^ A total of 68 children belonged to the families, 20 were not included in the baseline questionnaires for different reasons: <6 years, their condition, or choosing not to participate in the questionnaires. ^3^ Definitions of life-limiting and life-threatening conditions from [5].

**Table 3:** Intervention effects based on estimates from the linear mixed-effects models.

	Time Point	*n*	Mean (SD)	Estimate (SE) ^a^	95% CI	*p*-Value
Family Communication Scale (FCS)	Baseline	79	36.9 (6.05)	Ref.		
	Follow-up 1	66	38.1 (6.73)	1.16 (0.58)	0.01, 2.31	0.049
	Follow-up 2	48	39.2 (5.68)	1.9 (0.65)	0.61, 3.19	0.005
Family Satisfaction Scale (FSS)	Baseline	79	32.6 (6.7)	Ref.		
	Follow-up 1	65	34.2 (6.4)	1.41 (0.57)	0.3, 2.53	0.014
	Follow-up 2	48	35.3 (5.8)	2.57 (0.64)	1.32, 3.82	<0.001
Parental Skills Checklist total (PSC)	Baseline	53	28.1 (10.9)	Ref.		
	Follow-up 1	47	29.6 (11.7)	2.57 (1.49)	−0.37, 5.48	0.088
	Follow-up 2	38	30.8 (10.4)	3.44 (1.6)	0.28, 6.56	0.035
Elicitation Skills Subscale (PSC-ES)	Baseline	53	8.05 (3.04)	Ref.		
	Follow-up 1	47	7.94 (3.1)	0.18 (0.36)	−0.54, 0.89	0.631
	Follow-up 2	38	8.55 (2.8)	0.67 (0.39)	−0.1, 1.43	0.092
Connection Coping Skills, Subscale (PSC-CC)	Baseline	53	20.1 (8.29)	Ref.		
	Follow-up 1	47	21.6 (8.98)	2.37 (1.19)	0.00, 4.69	0.051
	Follow-up 2	38	22.3 (7.84)	2.74 (1.28)	0.22, 5.24	0.035
Generalized Anxiety Disorder Scale (GAD7)	Baseline	64	8.31 (5.22)	Ref.		
	Follow-up 1	56	6.45 (4.62)	−2.0 (0.57)	−3.12, −0.88	<0.001
	Follow-up 2	42	5.81 (4.6)	−2.38 (0.63)	−3.61, −1.13	<0.001
Resilience Scale 14 (RS14)	Baseline	72	76.7 (11.4)	Ref.		
	Follow-up 1	60	77.6 (14.4)	1.45 (0.89)	−0.29, 3.82	0.105
	Follow-up 2	45	79.8 (9.39)	0.72 (0.99)	−1.2, 2.27	0.461
Resilience Scale 10 (RS10)	Baseline	21	33.9 (3.34)	Ref		
	Follow-up 1	20	34.2 (3.65)	0.26 (0.62)	−0.95, 1.48	0.678
	Follow-up 2	14	33 (3.86)	−1.01 (0.74)	−2.47, 0.45	0.186
Strengths and Difficulties Scale (SDQ) Total difficulties	Baseline	38	10.8 (5.29)	Ref		
	Follow-up 1	33	9.24 (5.63)	−1.53 (0.7)	−2.90, −0.15	0.034
	Follow-up 2	21	9.62 (5.85)	−1.14 (0.84)	−2.78, 0.49	0.178
Strengths and Difficulties Scale (SDQ) prosocial	Baseline	38	8.5 (1.74)	Ref.		
	Follow-up 1	33	8.42 (2.12)	−0.1 (0.31)	−0.69, 0.49	0.744
	Follow-up 2	21	8.05 (1.77)	−0.49 (0.36)	−1.19, 0.21	0.177

^a^ Estimates are derived from a linear mixed-effects models using restricted maximum likelihood estimation (REML), with time specified as a fixed effect and individuals as random intercepts.

## Data Availability

Datasets from this study are available upon reasonable request. The data are not publicly available due to restrictions of privacy and for ethical reasons.

## Abbreviations

The following abbreviations are used in this manuscript:

FTI	Family Talk Intervention
MRC	Medical Research Council
FCS	Family Communication Scale
FSS	Family Satisfaction Scale
RS-14	Resilience Scale 14
RS-10	Resilience Scale 10
SDQ	The Strengths and Difficulties Questionnaire
PSC	Parental Skills Checklist
GAD-7	Generalized Anxiety Disorder
